# The Institutional Library

**Published:** 1919-11-01

**Authors:** 


### THE INSTITUTIONAL LIBRARY.

### An Evolutionist at Home.*

Great men usually dislike pretentious descriptions of themselves. The great artist is content to call himself a painter: the great dramatist, a playwright, as if but for an accident his job was to make carts instead of comedies. Butler would not describe himself a scientist, and was glad when during his life the term was forbidden to him by his more successful colleagues in that field. He knew that his business was to digest the information collected by experts in research, to think about facts, and to discern their relation to each other. His four books on Evolution are important to posterity in several respects. First, because evolution is the central theory from which spring the few modern ideas that we have; secondly, because he was concerned not merely with the theory and its clarification, but with its implications which necessarily illumine many problems of life; thirdly, because, being concerned with man's religious needs, he was the most ambitious of those who have attempted to build a philosophy of life upon evolution; fourthly, because he did succeed in making the theory of evolution more interesting and more clear to the average man than any of its later supporters.

### Four Books on Evolution.

Thus the four books on evolution define the simple generalisations (which we call laws of Nature), whereon he believed that the explanation of the process of evolution must rest. These generalisations are: the oneness of personality between parents and offspring; the memory in the offspring of the actions which it performed in the persons of its ancestors; the latency of that memory until awakened by association of ideas; and the unconsciousness with which habitual actions come to be performed. Instinct therefore means "inherited memory," and memory is the power to repeat sensations and actions, which in time become, first, habitual, then instinctive. When they have become instinctive, we have forgotten how it is that we are able to do them. By means of these generalisations Butler made the process of evolution more intelligible; and, since he was not a "scientist" but "a philosophical writer," he was also able to throw interesting lights upon the cause of longevity and to suggest how it might be extended; on the cause of the observed sterility of hybrids; and, lastly, to affirm a correspondence between the "laws" of evolution and similar "laws" in relation to the assimilation and growth of our ideas, our opinions, our legislation, and our friendships. This was the reward of the singular concentration of his mind and of the attention which he gave to that which primarily occupied it. Butler wrote with such care that, seventeen years after his death, we are tempted to predict that his ideas, like Wordsworth's yew-tree, were "produced too slowly ever to decay."

In himself he was a shy old bachelor with a rather grim sense of humour, who was known to few and passed through life almost unnoticed. His books, which only his uncertain private means enabled him to publish, were the chief events in a quiet but not unchequered life. He was from boyhood always methodical. He shows how rich are the rewards of the dull virtues—patience, attention, method, pains. He did for odds and ends of fact that which the Waifs and Strays Society does for other little ragamuffins: he picked them up and housed and fed them. All was docketed in his pocket-book, and though much was destroyed by himself as uninteresting, much remains. He wrote and received but few letters; but those which he wrote were treasured, and those which he received were kept. He had three friendships: one with a man whom he met when sheep-farming in New Zealand, after his Cambridge days were done—the man abused Butler's generosity; one with the dear, but ugly, old Miss Savage, who fell in love with him, but whom he felt no inclination to marry—as certain grim and tender posthumously published sonnets explain. The last long friendship was with Mr. Festing Jones, who has now written his life. Butler had also three enemies. The first was his father; the second, his mother; the third, Charles Darwin. Only the last of these unwelcome feuds arose from a misunderstanding which might have been prevented, and this Darwin's descendants have done their best to repair. Butler was much damaged by these bitternesses. They made his books, however excusably, polemical; they turned his native sweetness to acidity, as a hard frost will nip or embitter the promising fruit-blossoms of spring; they intensified his bachelor detachment; they made rather a hermit of a man who was not more inclined by nature to seclusion than anyone is who knows, because he cares for thought, that good thoughts do not come from thinking so much as from remembered observation and the scholar's quiet. He was naturally shy; he became reserved. He was naturally silent; he became moody. He was naturally witty; he became satirical. To this did his family reduce him. Yet of this shy, retiring, warm-hearted but wounded man we now know more than of any Englishman except that other Samuel, Dr. Johnson—a man, by the way, who was "a philosophical writer" also, and our first "scientist" in the domain of lexicography. For Butler's methodical care in his life and Mr. Festing Jones' methodical care after his death have achieved that which Boswell achieved by a methodical, but blind, devotion. Mr. Festing Jones' Memoir is an extraordinary presentation. It is the latest instance how and why the tortoise really won the race.

### The Man Himself.

We are shown not so much a great man, though Butler was great enough to have great limitations, but a man known as he was known to himself, to his friends, to his servant, and by report both at home and abroad; in his rooms, and as an author. We are shown, too, that the "secret" of greatness is too simple to be believed, except in its fruits, by most people, and how a measure thereof can be attained by anyone who admires the real thing enough in others to understand and so to imitate their example, of care in method and of purpose in simplicity of choice. We see also how it comes about that the same character may combine such different qualities as sunniness and acerbity, grimness and humour; and can measure the effect of denying responsiveness to a responsive child, an effect which is, when repression is rigorously pursued, to numb it so severely that any subsequent mellowing is a miracle.

If Butler had not combined with his insight into fact and character a sense of humour, which he learnt to

* *Samuel Butler: Author of "Erewhon."* A Memoir by Henry Festing Jones. (London: Macmillan and Co. Price 42s. net.)

**The Institutional Library**—(*continued*).

reverence, and thus vented the accumulated bitterness insinuated like a poison into him at home, he would have been a sour apple to the end. As it was, he mellowed: one side at least remained warmed by the sun; but his humour is often harsher than it would have been had his parents not persecuted him with remorseless stupidity. His mother, for instance, in one of the customary "religious jaws" to which she subjected him in boyhood, begged him, with tears in her eyes, to "have his loins girt about with the breast-plate of purity"; and these typical and persistent precepts twisted his moral nature as much as the breast-plate would have damaged his legs. The book, too, is full of good stories; a pleasant levity pervades it, and these stories are of the genuine kind which most of us chance on only to forget. In print, therefore, they possess a freshness which is that of a characteristic sally in a private letter from a friend, a freshness that can hardly stale, for each story is left in its original soil of character, and scene, correspondence, or company. It is as impossible to select from such a store as it would be to pull a plum from a Christmas pudding. It is not a plum but a second helping that we want. Since Boswell was a conceited fool, with only admiration and method to equip him for the writing of a masterpiece, imagine the quality of a biography written by a methodical friend who is *not* a fool, and, unlike Boswell, in perfect sympathy with a no less interesting and prolific subject! Unlike Boswell, Mr. Festing Jones is not at pains to intrude his admiration. Like a good dramatist he leaves the applause to be inferred, with the result that we are so fascinated as to feel applause to be an impertinence in Butler's presence. It is the sense of his presence that lingers; and the conviction that Butler, for all his waywardness, was a great thinker on the science and art of life.

**Surgery of the Lung and Pleura.** By H. Morriston Davies, M.A., M.D., M.C.(Cantab.), F.R.C.S.(Eng.). (London: Shaw and Sons, 7 and 8 Fetter Lane, E.C. 4. Price £1 5s. net.)

This work we can fully recommend to those who would possess an up-to-date and reliable reference book upon the subject of thoracic surgery. Within its pages are combined with singular conciseness the theoretical and practical aspects, and in spite of this necessary process of condensation the excellent manner and quality in which the book has been produced make it welcome reading to the surgeon and student alike. It must be realised that very great advances have occurred since 1903, when surgeons first began successfully to work out methods of preventing collapse of the lung after free opening of the pleura. Achievements then recorded have led first to considerable extension of the measures possible of performance on this part of the body and later to great simplification of the technique employed in what many of us may still view as operations of great difficulty and risk. Mr. Morriston Davies' book thus opportunely introduces the reader to the true facts relating to thoracic surgery as it stands to-day. As he explains, study of the special mechanical conditions which obtain in the thorax, the use of gases within the pleura to obtain on the one hand immobilisation of the lung and on the other hand the replacement of effusions under control by the observations of pressure and by radiography; and a very large body of experience in chest injuries derived from the war—all these have contributed in their degree to rapid development and modification and to the need of a general review of the present state of the whole subject. The review he provides is certainly well worth our attention. Particularly excellent is his chapter on pulmonary tuberculosis, where the modern theories as to routes of infection and diagnosis of the "early case" are followed by a clearly demonstrated summary of the means of treatment now available. Nitrogen displacement (artificial pneumothorax), the arrest of hæmorrhage, the treatment of adhesions, rib mobilisation, local displacement by foreign bodies, paralysis of diaphragm by section of the phrenic nerve, are all given full and practical consideration. The volume is profusely illustrated with excellent radiograms, coloured plates, and descriptive charts, and throughout provides what, in a highly technical subject, is even more valuable—namely, opportunity to obtain a clear knowledge and a practical understanding of it.

**Cancer Research and Vivisection.** By D. Macmillan. Price 1s.

A copy of this pamphlet, which emanates from a source entitled the "Society for the Prevention and Relief of Cancer," has been forwarded to us. The chief argument developed is that cancer research by vivisection has failed so far to help much in controlling that disease; a statement which this Journal made some years ago (June 22, 1912, p. 287), and one that is still true. From this to the prohibition of all vivisection is a leap which Mr. Macmillan does not actually take, though apparently he would like to. Above and beyond this the "Society" which issues the pamphlet appeals for funds. Upon which it is only necessary to remark that no information as to its officers, members, or promoters is published, except the statements that it consists mainly of laymen and that the Duchess of Hamilton is a patroness. As Mr. Macmillan sees fit, in an appendix, to attach enormous importance to the views of various medical men, of whom it is true to say that several of them have incurred the suspicion and distrust of the medical profession, it is perhaps not unfair to assume that this "Society" is not one that can be recommended to public support.

**Defective Housing and the Growth of Children.** By J. Lawson Dick, M.D., F.R.S. Allen and Unwin. Pp. 94. Price 3s. 6d. net.

Dr. Dick's thesis is that rickets is much less a disease of faulty nutrition than a disease of faulty sanitation, chiefly of insufficient air and light. He criticises with considerable success the two current hypotheses that rickets is due to lack of vitamines in the infant's food, or that it is due to neglect of breast-feeding. The whole brochure is interesting and well arranged. Our only criticism is that it could, without curtailment by a single word, have easily been printed on forty pages instead of ninety-four, and could then presumably have been marketed a good deal cheaper than the price at which it is issued.

**Hospital Sketches.** By Frances Lyndall. (London: George Allen and Unwin, Ltd. Price 2s. net.)

This collection of fourteen sketches, presumably by a nurse, is a slight record of hospital experience, some of which appears to have taken place in France or Italy. They are records rather of the young lady's impressions of her life than of things seen and remembered, and only in "Our Italian Orderly," the best of the series, and in "The Protégé of a Princess" is there any attempt at character studies. There is less humour and more sentiment than will be agreeable to all tastes, but the little book is honest through its limitations, and probably presents a fair picture of the impressions left by a military hospital on the mind of a young V.A.D. nurse. As such it is an index of a large and enthusiastic class which has made its first acquaintance with the rough-and-tumble of the world through nursing during the war.

